# Giant vaginal lithiasis in spinal cord injury related neurogenic lower urinary tract dysfunction: diagnostic pitfall with upper urinary tract deterioration

**DOI:** 10.1038/s41394-026-00745-1

**Published:** 2026-08-11

**Authors:** Tudor-Mihai Magdaș, Bogdan Adrian Buhas, Sofia Anamaria Ferțadi, Dan Vasile Stanca

**Affiliations:** 1 Department of Urology, Clinical Municipal Hospital, 11 Tabacarilor St., 400139 Cluj-Napoca, Romania; 2 “Iuliu Hațieganu” University of Medicine and Pharmacy, Cluj-Napoca, Romania

**Keywords:** Neurological manifestations, Neurogenic bladder, Urinary tract infection, Risk factors

## Abstract

**Introduction:**

Vaginal lithiasis is exceptionally rare and may mimic vesical lithiasis on imaging, particularly in women with spinal cord injury (SCI) and neurogenic lower urinary tract dysfunction (nLUTD). Delayed recognition may cause recurrent infection, obstructive uropathy and upper urinary tract deterioration.

**Case presentation:**

A 38-year-old woman with a T6 ASIA Impairment Scale (AIS) grade A SCI and nLUTD after epidural abscess drainage presented with continuous urinary leakage, recurrent febrile urinary tract infections and progressive renal impairment. Urine culture grew *Pseudomonas aeruginosa*. Non-contrast-enhanced computed tomography showed a 10 × 5.7 cm midline pelvic calculus with bilateral grade II–III ureterohydronephrosis, initially read as a giant intravesical stone. Persistent incontinence was inconsistent with presumed detrusor–sphincter dyssynergia, prompting clinical reassessment. Cystoscopy revealed an empty bladder and a midline vesico-vaginal fistula; colposcopy confirmed a giant vaginal calculus occupying the vaginal vault. The calculus was removed transvaginally using Kielland obstetric forceps. Hydronephrosis resolved and renal function improved; fistula repair was deferred per patient preference.

**Discussion:**

A focused review identified fewer than 20 reported cases of primary vaginal lithiasis, most associated with nLUTD, neurological impairment, anatomical abnormalities, urinary fistulas or chronic urinary stasis. This case emphasizes that imaging alone may mislocalize pelvic calculi. In SCI patients with nLUTD, clinical and pelvic examination are essential and may establish the diagnosis while assessing for fistula. After risk stratification, structured long-term surveillance of the upper urinary tract and awareness among spinal cord–injured individuals and physicians are crucial to prevent complications and recurrence.

## Introduction

Vaginal lithiasis, also referred to as vaginal calculus or colpolithiasis, is a rare and underreported condition that poses significant diagnostic and therapeutic challenges. Typically described in isolated case reports, this entity is often misdiagnosed due to its low incidence and nonspecific symptomatology, frequently being mistaken for vesical lithiasis, pelvic masses, or foreign body complications [1–3]. Although the precise prevalence is unknown, the limited number of documented cases suggests that most gynecologists and urologists may never encounter a case during their clinical practice [1]. Patients with spinal cord injury (SCI) and neurogenic lower urinary tract dysfunction (nLUTD) are recognized to have a significantly elevated risk of upper urinary tract deterioration, including hydronephrosis, urinary fistulas, lithiasis, and progressive renal impairment, necessitating lifelong urological monitoring and structured follow-up [4]. This need is further supported by risk-prioritization frameworks proposed by Hentzen et al., where spinal cord injury patients with upper tract involvement, recurrent urinary tract infections (UTIs), and fistulas are classified as requiring urgent evaluation and intervention [5].

Vaginal lithiasis is broadly classified into two types based on pathogenesis: primary and secondary. Primary vaginal lithiasis develop de novo in the absence of foreign bodies and are most often associated with chronic urinary stasis in the vaginal cavity. This stasis may result from congenital anomalies, nLUTD, vesico-vaginal or urethro-vaginal fistulas, vaginal outlet obstruction, or prolonged immobilization, especially in neurologically impaired patients [2, 3, 6]. In contrast, secondary vaginal lithiasis form around a nidus, such as a forgotten pessary, surgical gauze, intrauterine device, or other retained foreign bodies, and may also occur following trauma or surgery [7, 8].

Several authors have emphasized the role of urease-producing bacteria (e.g., *Proteus mirabilis, Pseudomonas aeruginosa*) in stone formation, which alter the vaginal pH and promote precipitation of ammonium magnesium phosphate (struvite) crystals [3, 9]. In many cases, such stones grow slowly over several years, with symptoms ranging from malodorous vaginal discharge and chronic pelvic discomfort to urinary tract infections and, in rare instances, obstructive uropathy with hydronephrosis [1, 3, 10].

Despite the anatomical proximity of the vagina and bladder, the differentiation between vaginal and bladder lithiasis may be difficult on imaging, especially when large stones distort pelvic anatomy. Diagnostic delays are common, and appropriate identification often requires endoscopic or gynecological evaluation [1].

The diagnostic course, management strategy, and clinical outcome of the present case are described, together with a focused review of the available literature.

## Case report

We present the case of a 38-year-old woman with a history of severe neurological impairment who was referred to the urology department for evaluation of recurrent febrile UTIs and progressive renal dysfunction. The patient had sustained a SCI at the age of 22 following surgical drainage of a spinal epidural abscess, with irreversible sequelae including spastic paraplegia and chronic nLUTD. Initially managed with indwelling catheterization, the patient transitioned to the use of absorbent urinary pads six years prior to presentation due to persistent urinary incontinence. Over the preceding two years, she reported multiple episodes of high-grade fever associated with UTIs, managed empirically in outpatient settings. Her neurological status was documented with reference to the ASIA/ISCoS International Standards for Neurological Classification of Spinal Cord Injury (ISNCSCI) [11]; at the most recent assessment the neurological level of injury was T6 and the ASIA Impairment Scale (AIS) grade was A, corresponding to a motor- and sensory-complete lesion, consistent with chronic spastic paraplegia.

On clinical examination, the patient was afebrile, paraplegic, and had continuous urinary leakage. Detrusor–sphincter dyssynergia (DSD) was the presumed mechanism; however, DSD typically causes retention and voiding difficulty, whereas her persistent dribbling was atypical and warranted further evaluation, underscoring why a thorough clinical and pelvic examination is indispensable when assessing incontinence in neurological patients.

Laboratory evaluation demonstrated leukocytosis, normocytic anemia, and impaired renal function (reduced estimated glomerular filtration rate). Urinalysis revealed pyuria and bacteriuria, and urine culture identified *Pseudomonas aeruginosa*. Empirical intravenous antibiotic therapy with ceftazidime was initiated.

**Fig. 1:**
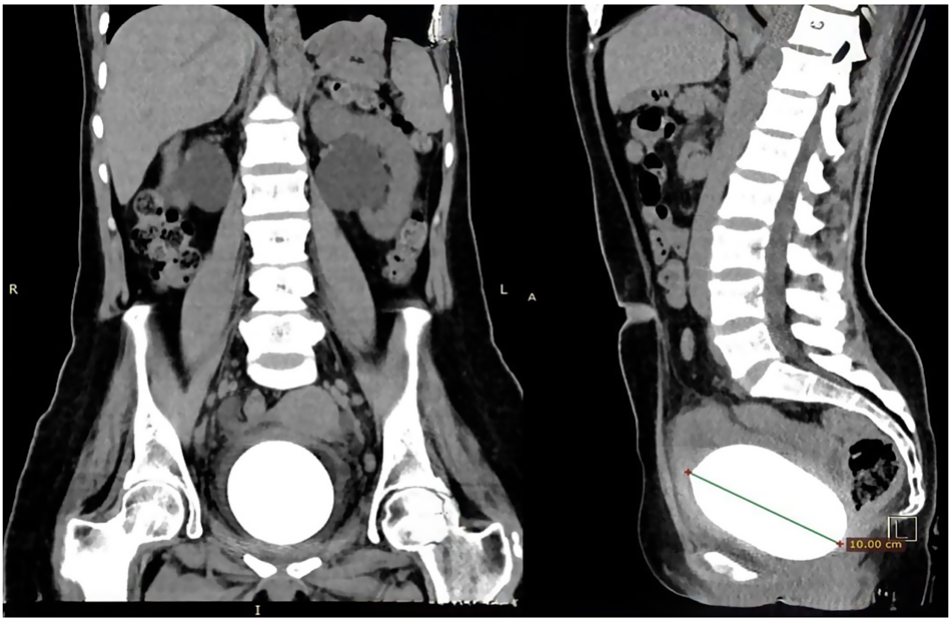
Coronal and sagittal NCCT scan revealing a giant 10 × 5.7 cm pelvic calculus and bilateral hydronephrosis.

Imaging studies were notable for bilateral moderate-to-severe hydronephrosis and a large calcified pelvic mass. Non-contrast enhanced computed tomography (NCCT) revealed a dense, oval-shaped calculus measuring 10 × 5.7 cm located in the midline pelvis, along with bilateral grade II–III ureterohydronephrosis (Fig. 1). The initial radiological interpretation favored a diagnosis of a giant intravesical stone causing bilateral ureteral obstruction and obstructive uropathy.

However, the clinical presentation was discordant with the imaging findings. The presence of continuous urinary incontinence in a patient with presumed DSD and a suspected obstructive bladder stone raised concern for an alternative diagnosis. To resolve this discrepancy and define the anatomical relationships, a diagnostic urethrocystoscopy was performed under general anesthesia, with appropriate precautions taken to prevent autonomic dysreflexia.

**Fig. 2:**
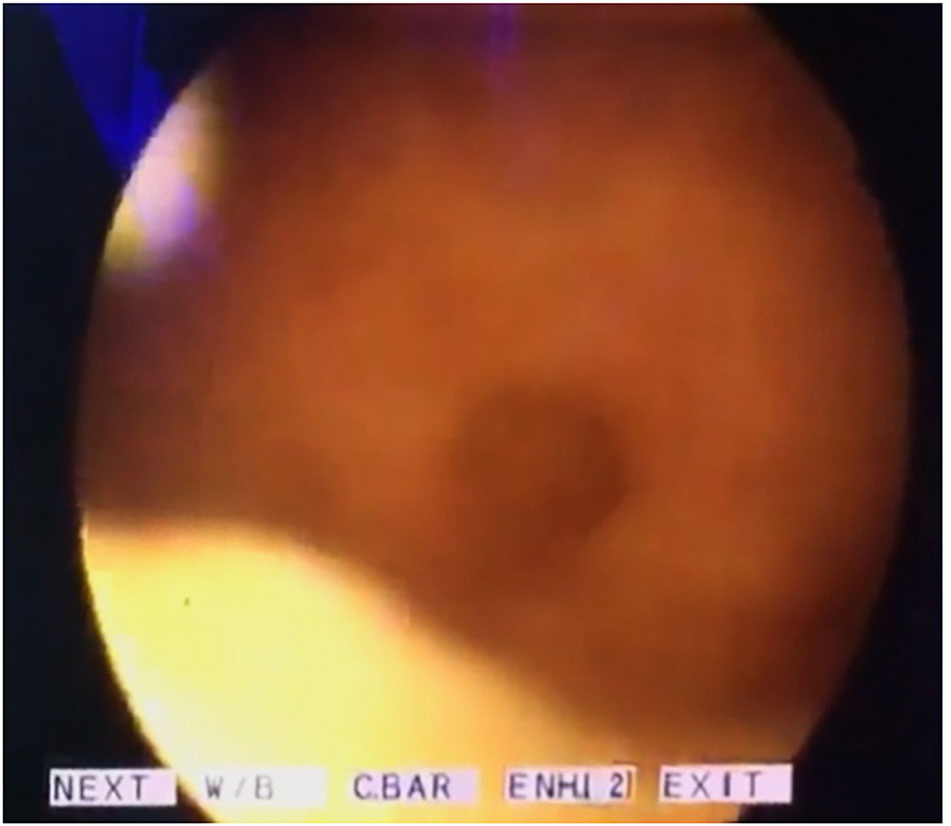
Intraoperative cystoscopic view revealing a vesico-vaginal fistula approximately 1 cm in diameter.

Cystoscopy revealed an unexpectedly empty bladder, with significant inflammatory changes of the postero-inferior bladder wall and obscured visualization of the ureteric orifices. A 1 cm midline vesico-vaginal fistula was identified posterior to the trigone (Fig. 2). Subsequent gynecologic evaluation via colposcopy confirmed the presence of a giant vaginal calculus occupying the entire vaginal vault.

**Fig. 3:**
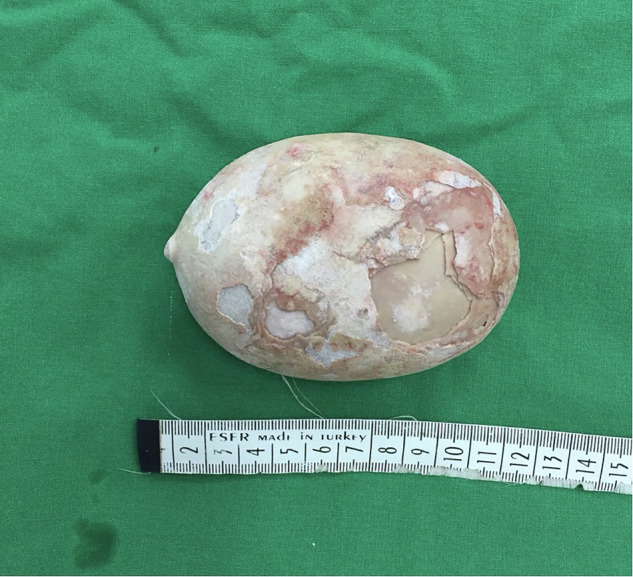
The extracted vaginal stone, measuring 10 × 5.7 cm.

Under general anesthesia, the stone was extracted transvaginally using Kielland obstetric forceps. The calculus measured 10 × 5.7 cm and displayed a laminated, irregular morphology consistent with prolonged mineral deposition (Fig. 3).

Following successful stone removal, a multidisciplinary consultation involving urology, gynecology, and the patient was convened to determine further management. Given the patient’s stable condition, lack of obstructive symptoms post-removal, and personal preference to avoid further invasive procedures, a conservative approach was selected. Surgical closure of the vesico-vaginal fistula was deferred, and the patient elected to continue managing urinary incontinence with absorbent products rather than intermittent catheterization.

Postoperative recovery was uneventful. Follow-up imaging demonstrated complete resolution of bilateral hydronephrosis with significant improvement in renal function, and vesicoureteric reflux was excluded. Urodynamic evaluation demonstrated neurogenic detrusor overactivity with detrusor–sphincter dyssynergia, confirming the suprasacral pattern of nLUTD expected for a T6 AIS A lesion. At the latest follow-up, the patient remained clinically stable, afebrile, and free of recurrent UTIs.

### Differential diagnosis

In neurologically impaired women with persistent urinary incontinence or pelvic masses, differential diagnoses include vesico-vaginal fistula, pelvic organ prolapse, vaginal neoplasms, urethral diverticulum, and retained vaginal foreign bodies [7, 8, 10, 12, 13]. Failure to consider these possibilities may delay diagnosis and contribute to complications such as upper urinary tract deterioration. In our case, malignancy was considered but excluded based on imaging characteristics and clinical context. No evidence of pelvic organ prolapse or neoplastic invasion was found on pelvic examination and NCCT. Given the rarity of primary vaginal lithiasis, a high index of suspicion is required, particularly when standard imaging suggests a radiopaque pelvic mass with irregular calcifications near the vaginal canal.

## Discussion

The present case adds several clinically relevant observations to the limited case-based literature on vaginal lithiasis. As summarized in Table 1, reported cases cluster around three main predisposing settings: severe neurological impairment or immobility [1–3, 9, 10, 12–17], anatomical abnormalities or genitourinary fistulas [6, 7, 18–20], and chronic infection-related urinary stasis with frequent struvite or mixed infection-stone composition [1, 2, 6, 9, 12–15, 17, 21]. Compared with most published cases, our patient is notable for the severity of upper urinary tract involvement, with bilateral grade II–III ureterohydronephrosis, recurrent febrile UTIs, and renal impairment. Hydronephrosis or obstructive upper-tract complications have been reported only in selected cases [1, 15, 17], emphasizing that vaginal lithiasis in women with SCI and nLUTD can progress beyond a local vaginal condition and become a threat to renal function.

**Table 1:** Representative reported cases of vaginal lithiasis associated with neurological, functional, anatomic, or fistula-related risk factors.

Author (Year)	Age (years)	Aetiology / predisposing factor	Stone size (cm) / weight	Composition / microbiology	Upper-tract involvement	Treatment	Outcome
Lin et al. [10]	43	Cerebral palsy (bedridden)	10 × 8 × 4.5; 935 g	Laminated calcification, fibrin	NR	Laparotomy + anterior vaginal incision	Uneventful recovery
Liu et al. [6]	14	Urethrovaginal fistula + vaginal stenosis	Multiple (5 calculi)	Triple phosphate (struvite)	NR	Transabdominal removal + fistula management	Recovery (3-mo follow-up)
Ho & Lin [7]	24	Congenital vaginal septum; anorectal malformation	4 × 3 × 1 & 2.1 × 0.8 × 0.4	Carbonate apatite	NR	Transvaginal extraction (ring forceps)	Recovery
Jaspers et al. [13]	5	Infantile encephalopathy, epilepsy	3 × 2 & 2 × 1	Struvite (MAP)	No	Nephroscope + ultrasonic lithotripsy	Recovery
Avsar et al. [2]	22	Paraplegia	9 × 7 × 2; 136 g	100% struvite; *E. coli*	No (fistula excluded)	Episiotomy + ring forceps	Symptom-free at 6 months
Castellan et al. [1]	34	Tetraplegia (West syndrome)	5.1 × 3.7 × 3.1; 156 g	100% struvite	Left hydronephrosis	Vaginal extraction (forceps)	Complete recovery
Tokgöz et al. [12]	14	Neurodegenerative disorder (bedridden)	3.8 (max)	Struvite; *P. aeruginosa*	No	Surgical removal (general anaesthesia)	No recurrence at 1 year
Aronson et al. (2020) [9]	54	Multiple sclerosis (nLUTD)	Large (NR)	70% struvite, 30% apatite	No (fistula excluded)	Surgical removal	Uneventful
Fedrigon et al. [14]	61	Cerebral palsy (bedridden)	≈11 × 9 × 8	Struvite (60% MAP, 40% CP)	No	Nephroscope + endoscopic ultrasonic lithotrite	Complete removal
Xu & Zou [18]	23	Urogenital sinus anomaly	7.8 × 6.8 × 7.7	Not specified	No	Vaginoplasty + urethroplasty	Full recovery
Noegroho et al. [19]	57	Urethrovaginal fistula (post-caesarean)	9.0 × 7.0	NR	NR	Transabdominal removal + hysterectomy + fistula closure	No leakage at 2 mo
Kurmanov et al. [20]	69	Vesico-vaginal fistula (uterine amputation + radiotherapy)	Medium (NR)	NR	NR	Transvaginal lithotomy + fistula repair	No recurrence
Owa et al. [15]	47	Cerebral palsy (bedridden)	8.1 × 5.6 × 7.0	Struvite (77% MAP, 23% CP); *P. aeruginosa*	Left hydronephrosis	Rigid cystoscope + pneumatic lithotripsy	Complete removal
Aslan et al. [16]	Adult (NR)	Spastic paraplegia	Huge (NR)	NR	NR	Surgical extraction	NR
Jo et al. [3]	28	Spastic quadriplegia (bedridden)	Large (NR)	100% struvite	No (fistula excluded)	Transvaginal removal via stump + hysterectomy	No recurrence at 3 months
Young et al. [21]	46	Cerebral palsy (nonverbal, lifelong paralysis)	11 × 10.5 × 10	Struvite	Right pelvicalyceal dilatation (14 mm)	Transvaginal hollow-out drilling	Complete removal
Chamma et al. [17]	27	Cerebral palsy (bedridden)	9 × 10	Mixed Ca-oxalate + struvite; *P. aeruginosa*	Obstructing ureteral stone + hydronephrosis	Nephroscope + ultrasonic lithotripsy	Uneventful recovery
Present case	38	T6 AIS A SCI (nLUTD) with vesico-vaginal fistula	10 × 5.7	Infection stone (presumed struvite); *P. aeruginosa*	Bilateral grade II–III ureterohydronephrosis (resolved)	Transvaginal extraction (Kielland forceps)	Hydronephrosis resolved; renal function improved

Primary vaginal lithiasis form de novo from urinary stasis without a foreign-body nidus; secondary lithiasis (forming on surgical mesh, intrauterine devices, sutures or stents) are not included. Composition is reported as published; “infection stone (presumed)” denotes a clinically infective (urease-associated) stone not submitted for formal crystallographic analysis.

*MAP* magnesium ammonium phosphate (struvite), *CP* calcium phosphate, *SCI* spinal cord injury, *nLUTD* neurogenic lower urinary tract dysfunction, *NR* not reported.

A central diagnostic lesson is that imaging alone may mislocalize a large pelvic calculus. In the present case, NCCT correctly identified a large midline pelvic stone but initially suggested an intravesical location. Similar diagnostic ambiguity has been described in previous reports, particularly when large stones distort pelvic anatomy, when the bladder is decompressed, or when a fistula or anatomical abnormality is present [1, 3, 6, 13, 19, 20]. The decisive clue in our patient was clinical–radiological discordance: continuous urinary leakage was not fully explained by presumed DSD mediated functional outlet obstruction. This prompted cystoscopy, which demonstrated an empty bladder and a midline vesico-vaginal fistula, while gynecologic assessment confirmed that the stone occupied the vaginal vault. Therefore, in neurologically impaired women, urinary incontinence should not be automatically attributed to nLUTD alone. Clinical and pelvic examination remain essential and may establish the diagnosis rapidly while also assessing for fistula, foreign body, vaginal obstruction, prolapse, urethral diverticulum, or malignancy.

The pathogenesis in this case was most consistent with chronic infection-related mineral deposition within the vagina. The vesico-vaginal fistula allowed persistent urinary pooling, while immobility, impaired pelvic sensation, nLUTD, and recurrent infection created favorable conditions for progressive stone growth. Several cases in Table 1 report struvite or mixed infection-related composition, and *Pseudomonas aeruginosa* has been documented in association with vaginal lithiasis in neurological or immobile patients [12, 15, 17]. This patient also had *Pseudomonas aeruginosa* bacteriuria, supporting an infection-related mechanism. However, because formal crystallographic analysis of the extracted calculus was not performed, the stone should be described as a presumed infection-related calculus rather than definitively struvite.

Management should be individualized according to stone size, location, vaginal access, local tissue condition, infection status, neurological comorbidity, anesthetic risk, and the presence of a genitourinary fistula. The cases summarized in Table 1 illustrate the range of reported approaches: transvaginal extraction with obstetric or surgical forceps, with or without episiotomy [1, 2, 7, 16]; transabdominal or open extraction for very large, adherent, or anatomically complex stones [6, 10, 19]; transvaginal hollow-out drilling for an impacted calculus [21]; and minimally invasive endoscopic fragmentation using cystoscopic, nephroscopic, ultrasonic, or pneumatic lithotripsy instruments [13–15, 17, 22]. In fragile neurological patients, vaginal or endoscopic approaches may avoid laparotomy and reduce morbidity. In this case, complete transvaginal extraction with obstetric forceps was feasible and led to resolution of hydronephrosis and improvement in renal function.

The coexistence of a vesico-vaginal fistula requires a separate management decision. Some authors have reported staged management, with stone removal followed by delayed fistula repair after infection, edema, and local inflammation have resolved [19]. Others have described concurrent stone extraction and fistula repair when local conditions are favorable and active infection is absent [20, 23]. In the present case, fistula repair was deferred after multidisciplinary discussion and shared decision-making. This decision reflected the patient’s preference to avoid further invasive treatment, the presence of active infection, the chronicity of her nLUTD, and the need to balance continence goals against quality of life, autonomy, and upper urinary tract safety. Conservative fistula management should not be considered definitive anatomical correction; it requires structured follow-up to ensure that urinary drainage remains safe and that recurrent urinary pooling, infection, or stone formation does not occur [24].

This case also reinforces the importance of risk stratification in nLUTD. Current neuro-urology guidance recommends individualized follow-up, regular upper-tract assessment, renal function monitoring, and baseline and periodic urodynamic evaluation according to risk category [4]. Within the Hentzen risk-prioritization framework, the combination of vesico-vaginal fistula, recurrent febrile UTIs, and bilateral hydronephrosis places this patient in a high-risk category requiring proactive surveillance rather than reactive treatment of complications [5]. Long-term management should therefore focus on upper urinary tract protection, safe bladder storage and drainage, optimization of bladder-emptying strategy where feasible, treatment of symptomatic infections, control of neurogenic detrusor overactivity when present, and reduction of urinary stasis.

A further question is whether her bladder management could be optimized. Absorbent pads achieve social continence but neither ensure low-pressure storage nor prevent the urinary stasis that drove stone formation. Given confirmed detrusor overactivity with dyssynergia, she is a candidate for clean intermittent self-catheterization combined with antimuscarinic or beta-3 agonist therapy, with onabotulinumtoxinA detrusor injection if overactivity proves refractory. Adherence to catheterization depends on education, caregiver support and continuity of care, and is consistently better under specialized follow-up. Structured lifelong rehabilitation and neuro-urology review, rather than episodic complication-driven presentations, would plausibly have improved compliance, allowed earlier detection of the fistula and possibly prevented this calculus. The patient has since been re-engaged with both services, with delayed fistula repair and a change in bladder management under discussion.

The limitations of this report should be acknowledged. First, it describes a single rare case, and management decisions cannot be generalized to all patients with SCI, nLUTD, or vesico-vaginal fistula. Second, the literature review is case-based rather than systematic; the published cases are heterogeneous regarding age, neurological status, anatomical abnormality, stone size, microbiology, stone composition, operative approach, and follow-up. Third, stone composition in the present case was not confirmed by formal crystallographic analysis. Finally, because fistula repair was deferred, long-term monitoring is required to assess the durability of upper-tract recovery and the risk of recurrent infection or lithiasis.

## Conclusion

Vaginal lithiasis should be included in the differential diagnosis of neurologically impaired women with chronic urinary leakage, recurrent UTIs, and pelvic calcification. In SCI patients with nLUTD, persistent leakage that is not adequately explained by functional obstruction should raise suspicion for an anatomical abnormality such as a vesico-vaginal fistula or ectopic stone. A high level of awareness among SCI individuals and clinicians, combined with routine clinical and pelvic examination, early cystoscopic and gynecologic assessment when symptoms are incongruent, risk stratification, and structured long-term upper urinary tract surveillance, is essential to prevent diagnostic delay, recurrent stone formation, and renal injury.

## Data Availability

The clinical data supporting the findings of this case report were obtained from the internal records of the Municipal Hospital of Cluj-Napoca, Romania. Due to patient confidentiality, the raw data are not publicly available but may be shared by the corresponding author upon reasonable request and with institutional approval.
